# The Moderating Effect of Acculturation Strategies on the Relationship Between Newcomer Adjustment and Employee Behavior

**DOI:** 10.3389/fpsyg.2020.02117

**Published:** 2020-09-01

**Authors:** Confidence Hommey, Jianhong Ma, Lebbaeus Asamani, Priscilla Hanson

**Affiliations:** ^1^Department of Psychology and Behavioral Sciences, Zhejiang University, Hangzhou, China; ^2^Department of Education and Psychology, College of Education Studies, University of Cape Coast, Cape Coast, Ghana; ^3^Department of Human Resource, Ghana Psychology Council, Accra, Ghana

**Keywords:** integration, separation, socialization, turnover intentions, work-related anxiety, work-to-work employees, school-to-work employees

## Abstract

Acculturation begins when people find themselves in a cultural setting other than theirs, and to demonstrate acceptable behaviors, one of two strategies is adopted: adapting to the new environment or retain one’s own culture. On the basis of these two, four strategies have been proposed. The current article examined the moderation effect of two of these acculturation strategies, integration and separation, on the relationship between newcomer adjustment, and work-related anxiety and turnover intentions. The study was in two folds, the first explored the moderation effect among new employees in general, notwithstanding their immediate past working experience; and the second part separated the sample based on two criteria: those prior to their current role were working in another firm (work-to-work employees) and those who just come directly from school (school-to-work employees). The sample was made up of 250 employees who had spent not less than 6 months and not more than 12 months in their current role, drawn from the private banking and insurance firms in Ghana. The PROCESS analysis of the data revealed that integration moderated the relationship between newcomer adjustment and work-related anxiety and turnover intentions among all samples. Separation moderated the relationships in all cases, but for the relationship between newcomer adjustment and turnover intentions in study 1 and among school-to-work employees. The evidence from this article points to the fact that the acculturation strategy that newcomers adopt has an effect on the relationship between their level of adjustment and some organizational outcomes; however, a slight difference exists if their immediate past engagement is considered.

## Introduction

In today’s organizational environment, workers create their identity more by their skills rather than the organizations they work in. According to Cornelißen et al. (2007), this attitude, to a large extent, plays a role in workers’ decision to leave their current roles in dissatisfactory circumstances in search for alternative jobs. Increasing attention on the self rather than the organization comes with it employee movements within and between organizations. This further makes new employee socialization (onboarding) a key issue not only for firms but also for newcomers. As they go through career life, individual employees undergo socialization at many points, and organizations will have to be dealing with new employees more often because of elastic personnel needs. A number of studies have asserted that during the socialization process the new employees’ experiences at this stage could impact on work-related attitudes, performance, and the possibility of remaining or otherwise in the organization (Bauer et al., 2007; Saks et al., 2007). Considering the fact that a high turnover rate often has an effect on organizational performance and financial costs (Kacmar et al., 2006), employers do not have the luxury of time to wait for so many months for their new employees to settle down in their new organization by themselves (Chen and Eldridge, 2011). Therefore, substantial resources are invested to speed up their adjustment process (Goldstein and Ford, 2002). Thus, examining this process has important theoretical and practical implications (Bauer and Elder, 2006).

The assumption behind this heavy investment in newcomers’ socialization is that a sound start can help new incumbents adapt to their new environment, enhance task performance, improve job satisfaction, and attain a lasting effectiveness, whereas a poor start may have a long-lasting negative effect for both newcomers (anxiety, dissatisfaction, etc.) and organization development (low performance) (Katz, 1980; Holton, 2001). However, it becomes necessary to explore all the likely variables that interrupt this process, making it difficult not to achieve the desired outcome. In the presence of the headways made in the organizational socialization research, there still remain some knots that need to be tightened as far as the relationship between adjustment and organizational outcomes is concerned.

Previous socialization researches have examined the impact of socialization tactics on newcomer adjustment (Saks et al., 2007; Lu and Tjosvold, 2013; Leo et al., 2020) and have found a significant impact. The socialization tactics adopted by the organization is geared toward “processing” the new employee to adjust to the working environment, subsequently exhibiting approved organizational behavioral outcomes. Other studies have explored individual factors such as personality (Peltokorpi and Froese, 2012) and newcomer information seeking (Bauer et al., 2007) on newcomer adjustment and work-related outcomes. These studies focusing on individual factors were birthed out of the need to develop an alternative approach to understanding socialization (Gruman et al., 2006), considering the individual is an active agent (Griffin et al., 2000) in the process of socialization. What these studies have not explored is how newcomers psychologically meander through contrasting organizations’ accepted ways of work and the individual’s acquired working behavior. Further, more common in organizations are culturally diverse groups of employees (Oerlemans and Peeters, 2010) the newcomer will be interacting with on a daily basis. It becomes imperative to gain insight into the effect of newcomers decision to either find a balance between their acquired attitudes and that which is existing in the organization or maintain acquired behavior and ignore how members of the current organization interact and go about work in the event of contrast.

When individuals from different cultural settings come into contact with another one, the process of acculturation begins (Oerlemans and Peeters, 2010). To successfully navigate their way within this new environment, one of two strategies is adopted, adapt to the new setting or maintain one’s own culture. At the backdrop of this, the current study seeks to explore the influence of these strategies on the relationship between newcomers’ adjustment and selected work-related behaviors. The study proposes that the kind of strategy new employees adopt will moderate the relationship between their level of adjustment and work-related anxiety and turnover intentions. Another gab observed in previous studies is the collective treatment of newcomers without cognizance to their immediate previous environment (Yozgat and Güngörmez, 2015). The second part of this study split the respondents into those directly from school to work and those directly from a working role. This will serve as good base on which to develop an organizational socialization program that takes care of the different background-related influences.

## Acculturation and Organizational Socialization

Unarguably, acculturation is among the most frequently mentioned and studied constructs in ethnic psychology, sociology, and anthropology (Chen et al., 2008). Researchers in this domain usually include some measure of acculturation in their studies to explore differences within ethnic groups and to understand the relationship between acculturation and psychosocial adjustment and health (Veen, 2015; Yang, 2018; Frisco et al., 2019). It is often used to explain human adjustment—mostly adaption of immigrants (Oerlemans and Peeters, 2010). Acculturation can be explained from two perspectives: group and individual. At the individual level, which is the focus of this study, emphases are on individuals who have become members of a group other than their original group, working out how to live together, through negotiation in other to avoid conflict (Berry, 2005). Juxtaposing this to organizational socialization, which involves the process by which newcomers make a transition from organizational outsiders to insiders (Bauer et al., 2007), some similarities could be drawn. In organizational socialization, the newcomer gains knowledge about the new group and adjusts to new jobs, work groups, organizational processes, and culture in an attempt to be a better member (Saks et al., 2007). In both instances, two different ways of doing things clash, and the individual is expected to find ways of negotiating through these differences. In the former, the individual adopts one of four strategies proposed by John Berry, and the emphasis of this article is to explore the role of two of these strategies in the adjustment process under organizational socialization.

Further, acculturation is most often studied among individuals living in countries or places other than where they come from or were born: among immigrants, refugees, asylum seekers, and sojourners (e.g., international students, seasonal farm workers; Berry, 2006). It is in this light that the researchers introduce this theory among employees who have just arrived in organizations that they have not been associated with.

### Acculturation Strategies

Vital components of the acculturation process are found in three main areas, acculturation conditions, acculturation strategies, and acculturation outcomes (Arends-Tóth and Van de Vijver, 2006). Arends-Tóth and van de Vijver (ibid) noted acculturation conditions comprise both group- and individual-level factors—attributes of the host society, which could be seen in the form of perceived or objective discrimination; attributes of the community of origin, e.g., political context; attributes of the immigrants’ group, e.g., ethnic vitality; and personal characteristics, e.g., expectations, norms, and personality.

The second dimension Arends-Tóth and Van de Vijver (2003) noted was acculturation strategies: the manner immigrants prefer to relate to their place of settlement and their country of origin. According to Schwartz et al. (2010), cultural psychologists have come to the recognition that acquiring the values, beliefs, and practices of the host country does not automatically mean that the immigrants will do away with theirs. As such, in an attempt to find a balance between these two different ways of doing things, certain mental strategies are adopted. In a model developed by Berry (2005), the intersection of these two dimensions produces four acculturation strategies: assimilation—adoption of the host culture and discarding of home culture, separation—rejection of the host culture and retaining the home culture, integration—adoption of the host culture and retaining home culture, and marginalization—rejection of both host culture and home culture.

Based on the proposal by Berry, this study adopts two of the strategies, integration and separation, in the process of adjustment by new employees at the workplace. Integration is known to be often associated with favorable psychosocial outcomes (Coatsworth et al., 2005; David et al., 2009) and tends to lead to better adjustment as shown in new employees lower depression, job anxiety, and prosocial behaviors (Szapocznik et al., 1980; Schwarz et al., 2007; Chen et al., 2008). In the same vein, organizations engage the services of individuals partly because of their attitude toward work, which is to a large extent molded by their previous working environment and expect them to bring this to bear. Some other organizations when they take on new employees, especially fresh graduates, expect them to fully imbibe their working culture but should come with an extra touch. These form the basis for the choice of these acculturation strategies.

It is worth noting that investiture versus divestiture, as organizational socialization tactics, is different from the acculturation strategy of integration and separation. The former is an approach the organization adopts in “people processing” (Ashforth et al., 2007) during socialization. It involves a set of programs geared toward affirming the attributes of the newcomer (investiture) or stripping away the newcomers identity so that they are confronted with uncertainty (Van Maanen and Schein, 1979). Integration and separation, on the other hand, are not strategies of the organization. They are the means by which the individual relates to variables within the new environment and acquired behaviors as explained earlier. For instance, coming from an organization where hierarchy was key and autonomy was relatively non-existent into an organization where he is given the autonomy to operate because the firm wants to maximize his skill (investiture tactics), the individual decides to create a balance between observing hierarchy and enjoying autonomy. The individual would be said to have adopted the acculturation strategy of integration. It will be interesting for further studies to explore the relationship between these organizational socialization tactics and acculturation strategies.

The existence of cultural diversity in organizations is as a result of some employees holding on to their previous ways of working. Cultural diversity has been found to relate to important work outcomes (Oerlemans and Peeters, 2010) such as enhanced creativity and decision making (McLeod and Lobel, 1992; Watson et al., 2002). Employees who are able to maintain two different cultures are associated with high psychological adjustment (Chen et al., 2008) and positive attitudes (Wagstaff et al., 2020). On the other hand, some studies assert the presence of more than one culture in an organization has a detrimental effect (Kadam et al., 2020) on employee behavior such as performance (Singh et al., 2019). In a study conducted by Wagstaff et al. (2020) on the bicultural identity and cultural intelligence of employees, they found an increase in the positive attitudes of individuals with two cultural identities. Having employees with diverse culture seems to be a two-edged sword, which could be as a result of variations in team’s attitude, beliefs, and reaction toward diversity (Lauring and Selmer, 2011).

At the backdrop of this, the present study proposes that integration and separation as acculturation strategies will moderate the relationship between newcomer adjustment and employee behaviors such as work-related anxiety and turnover intentions. Once the newcomer has become conversant with the new role, has gained much confidence on the job (self-efficacy), and feels socially accepted, it will have an effect on their work-related anxiety and turnover intentions. However, this relationship will either be strengthened or weakened by the adoption of one of the strategies of acculturation, integration, or separation.

## Employee Behaviors

### Work-Related Anxiety

Previous studies have found a relationship between stress and adjustment (Rosenbusch et al., 2014), job change (Shaffer, 2000), and taking a new job (London, 1997). Allan et al. (1999) in their study attempted to assess newcomer socialization and stress. Evidence from their article showed mentoring, which is a socialization tactics used to help newcomers adapt, positively correlated with the amount of help in coping with stress. They further found the entire socialization process is related with work-induced stress. In the early stage of employment, adjustment is an important factor in reducing the amount of work-related anxiety produced in the newcomer. Nash-Wright (2011) pointed out that we all feel anxious in anticipation of an event, where one will be judged, and an instance is the early period in the lives of new employees. They are expected to show signs of their quality while trying to adjust to their new environment, and this can be an anxious moment for them. Immediately the adjustment process seems not to be going well; most are likely to believe that common situations faced are unsafe, subsequently triggering panic attack. “Anxiety is an emotional response to potential future threat or danger, eliciting symptoms of negative affective, somatic, behavioral, and cognitive components along a continuum based on intensity and duration” (Sartori and Singewald, 2019). A rise in the levels of just normal, non-clinical anxiety can distort employees’ attitudes, behavior, and even performance (Mortensen, 2014).

### Turnover Intentions

Over the years, concerns have been expressed over the disproportionate evidence of employee turnover and its related costs (Shaw et al., 2005). Relatively high turnover comes with significant replacement and recruitment costs and as such more likely to affect organizational performance (Holtom et al., 2008). Quite a number of studies have attempted to investigate the importance of retaining newcomers during their early stage in their new organization, considering the highest turnover rate is observed at the first 4 weeks on the job (Kennedy and Berger, 1994). Lundberg and Young (1997) retorted most turnovers happen within the early few months in the new environment. Kennedy and Berger, and Lundberg and Young addressed the need for maximum importance to be attached to turnover among new entrants and their socialization in their new organizations. In studies by Lam et al. (2002), Lo and Lam (2002), and Yang (2008), turnover intention among new employees was explored, with the intention that the process of organizational socialization has the tendency to result in intentions to leave and organizational commitment.

Kenexa Research Institute (2007), in a study conducted among multinational organizations in both the United States and United Kingdom, found out that 57% of new employees are inclined to quit their employment after less than 2 years on their job. O’Reilly et al. (1991) in studying new employees’ socialization attempt to draw a relationship between the person–organization fit and work outcomes. In this study, two variables, one of which is related to the current study, turnover intention, were studied. In their study, they found employees would have reduced commitment and a high level of intent to leave if they do not fit into the organization’s cultural requirements. The issue of turnover intentions becomes very much important to employers at the early stage of an individual’s entry into an organization not only because it is a direct predictor of turnover but also because organizations spend a considerable amount of time and other resources in recruiting and training the newcomer (Gao, 2011). Ongori (2007) asserts organizations invest so many resources in their new employees in the area of induction and training, developing, maintaining, and retaining them in their organization. As a major concern for organizations attempting to minimize financial loss that comes with training new employees, Ghadi (2017) noted that tackling factors that fuel employees’ intention to leave their jobs is prime. Studies on turnover intentions among new employees have been relegated to the background in recent times. And Gao (2011) noted that, for organizations to retain their employees, effective human resource practices, of which onboarding programs are a part, are to be adopted.

In summary, as cited by Lin et al. (2017), employees’ characteristics can be assigned geographically, where individuals’ domicile has a say in whom they are (Berry and Lampo, 2000; Allik and McCrae, 2004). Having spent most of their time in either school or another organization, they are molded by that environment and need to find a way of adapting to their new work setting, which often has somewhat different ways of behavior. Adjustment therefore becomes very important not only to the organization but also to the new employee. However, how the newcomers handle the conflict between their acquired work-related values and that of the organizations’ accepted way has been left unexplored. Acculturation theory, which basically looks at how one deal with two opposing cultures, becomes very useful in understanding newcomer adjustment and its associated outcomes. Based on this review, a model is proposed.

## Proposed Model

**FIGURE 1:**
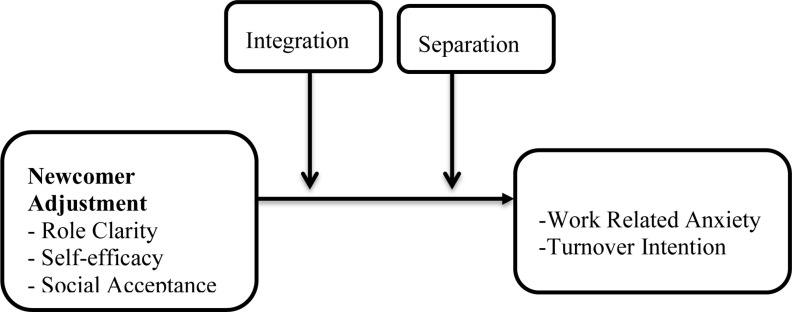
Proposed model of the study.

Figure 1 represents the relationships between the variables assessed in this study. This model was studied under two broad conditions. The first study explored this model among all employees not considering whether or not prior to their current role were from another organization [work-to-work employees (WtWEs)] or were directly from school (school-to-school employees). The second study then split the sample based on two criteria, school-to-work and work-to-work. This was to find out whether acculturation strategies moderated the relationship between newcomer adjustment and the behavioral outcomes being measured in these two different groups. Out of this framework, the following hypotheses were proposed.

### Hypotheses

#### Study 1

H1_a_: Integration will moderate the relationship between newcomer adjustment and work-related anxiety.
H1_b_: Separation will moderate the relationship between newcomer adjustment and work-related anxiety.
H2_a_: Integration will moderate the relationship between newcomer adjustment and turnover intentions.
H2_b_: Separation will moderate the relationship between newcomer adjustment and turnover intentions.

#### Study 2

H3_a_: Among WtWEs, integration will moderate the relationship between newcomer adjustment and turnover intentions.
H3_b_: Among WtWEs, separation will moderate the relationship between newcomer adjustment and turnover intentions.
H4_a_: Among WtWEs, integration will moderate the relationship between newcomer adjustment and work-related anxiety.
H4_b_: Among WtWEs, separation will moderate the relationship between newcomer adjustment and work-related anxiety.
H5_a_: Among school-to-work employees (StWEs), integration will moderate the relationship between newcomer adjustment and turnover intentions.
H5_b_: Among StWEs, separation will moderate the relationship between newcomer adjustment and turnover intentions.
H6_a_: Among StWEs, integration will moderate the relationship between newcomer adjustment and work-related anxiety.
H6_b_: Among StWEs, separation will moderate the relationship between newcomer adjustment and work-related anxiety.

## Procedure

Newcomers have been viewed as a subset of organizational members that have recently entered an organization (Rollag, 2010, p. 63). Most empirical studies focus the use of the term “new” on the length after organizational entry; thus, workers in the first 9 months or a year of employment in organizations are considered as new employees (Lam et al., 2002). For this study, the inclusion criterion in relation to length of time an employee has spent in current organization was not less than 6 months and not more than 12 months. The researchers considered the fact that for less than 6 months the new employee would still be in the early stages of adaption where induction programs are being organized for them and at more than 12 months most would forget what happened in their early stages of employment.

Further, two different categories of workers were considered as participants. First, employees who already have working experience and prior to their new role were working with a different firm or in a different role in the same organization, WtWEs. The second category of participants was employees coming into the organization directly from school, StWEs. The participants were employees working in different firms in the private sector in Ghana; specifically, five banking and seven insurance firms were conveniently sampled. The employees cut across the different departments in the firm. In total, 267 questionnaires were given to randomly sampled employees who had met the inclusion criteria from 12 different organizations. The researchers had an initial discussion with the human resource managers in the firms to identify staffs who met the inclusion criteria, after which participants were randomly selected, and the human resource managers/officers distributed the questionnaires at different times. However, in two of the firms, the staffs that meet the inclusion criteria were 11 and 13, and all of them were added to the participants. Of the 267 questionnaires distributed, eight employees did not return theirs, and nine questionnaires were less than 80% filled. The total number of sample whose responses to the questionnaires were used for this study was 250 employees, representing approximately 94% response rate.

## Data Analysis

All answered questionnaires used for the analysis were screened to make sure they were not less than 80% filled. To ensure data entered into SPSS reflected that which respondents provided, a different research assistant did a second check of the data by comparing responses on the questionnaires to the ones entered into SPSS. Descriptive statistics were computed for the demographic data. With regard to the hypotheses, analysis was performed following the procedure for moderation analysis expounded by Hayes (2013). For this study, there are two separate moderators, integration and separation, as such PROCESS constructed the necessary products, estimated the model, and generated the conditional effect of the independent variables on the dependent variables for various values of both moderators. The PROCESS model 2 is used for the analysis. This model is used when the effect of *X* on *Y* is influenced by 2 separate moderators, *M* and *W*.

## Instruments

### Newcomer Adjustment

Role clarity, self-efficacy, and social acceptance were the components of newcomer adjustment measured. Role clarity had four items measured on a five-point scale ranging from 1 (not clear) to 5 (very clear). Some of the items were as follows: *How clear are you about the limits of your authority in your present job? Do you feel you are always as clear as you would like to be about how you are supposed to do things on this job?* This scale was adopted from the work of Lyons (1971), which has been validated and used by different researchers over the years. The original estimate of Cronbach α for this scale was 0.70. However, for this study, Cronbach α for this scale was 0.85.

The measure used for self-efficacy was the General Perceived Self-efficacy scale developed by Scholz et al. (2002). The instrument was a 10-item scale measured on a five-point Likert scale ranging from 1 (not at all true) to 5 (exactly true). Some of the items were as follows: *I can always manage to solve difficult problems if I try hard enough*; *I am confident that I could deal efficiently with unexpected events*. The Cronbach α as computed in this study was 0.92.

The third and final scale used in measuring adjustment was social acceptance scale. The social acceptance scale was made up of three items measured on a five-point scale developed from the works of Bauer et al. (2007). It ranges from 1 (strongly disagree) to 5 (strongly agree). Some of the items were as follows: *I feel accepted in the informal groups at work*; *I have no problem with how my colleagues relate to me outside of work*. The Cronbach α for this scale for this study was a moderate one, 0.55.

### Integration and Separation

The instruments for integration and separation each contain four items measured on five-point scale. These items were developed based on an extensive review of acculturation measures by Arends-Tóth and Van de Vijver (2006) and the Acculturation, Habits, and Interests Multicultural Scale (Unger et al., 2002). They provided certain parameters and an in-depth explanation for the concepts. Based on this, six items for integration and five items for separation were developed. Using the dataset from this study, the reliability of the instrument was tested, and two items for integration and one item for separation had a very low correlated item-total correlation, and removing those items increased the Cronbach α of the instrument. After these items were removed, the Cronbach α’s for the instruments were 0.79 and 0.98 for separation and integration, respectively.

### Turnover Intention

A three-item scale measured on a five-point Likert scale developed by Mobley et al. (1978) was adopted for this study. The scale ranged from 1 (strongly disagree) to 5 (strongly agree). Some of the items in the scale were as follows: *I often think about quitting my present job*; *I will probably look for a new job in the next year*. From the dataset used for this study, the Cronbach α obtained for this scale was 0.88.

### Work-Related Anxiety

The Work Anxiety Scale developed by McCarthy et al. (2016) was used. The instrument contained eight items on a five-point Likert scale ranging from 1 (strongly disagree) to 5 (strongly agree). Some of the items in the scale were as follows: *I am overwhelmed by thoughts of doing poorly at work*; *I worry that my work performance will be lower than that of others at work*. The original scale had an internal consistency coefficient of 0.94. Using the data for this study, a Cronbach α of 0.968 was obtained.

## Results

### Study 1

Study 1 examined the moderating effect of integration and separation on the relationship between newcomer adjustment (independent variable) and work-related anxiety and turnover intentions (dependent variables) among all the participants put together. The purpose of this study was to explore the relationships between these variables among employees in general, notwithstanding their immediate past engagement. The researchers wanted to find out if all employees were treated the same, what would have been the outcome, and find out whether acculturation strategies would moderate the relationship between newcomer adjustment and work-related anxiety and turnover intentions. In order to check for the moderation effect, model 2 in PROCESS command in SPSS developed by Andrew Hayes was used for the analysis.

#### Demographics of Respondents

The number of valid response used for the analysis was 250, of which 58.8% were males (Table 1). All 250 responses used in the analysis met the inclusion criteria. The average number of months respondents had been in their current role was 8 months. In relation to the age range of the respondents, the majority (40.80%) of them were from the ages of 25–29 years.

**TABLE 1:** Demographic characteristics (study 1).

**Age**			**Sex**			**Months in current role**	
**Profile**	**Frequency**	**Percent**	**Profile**	**Frequency**	**Percent**		
18–24	60	24.00	Male	147	58.80	Minimum	6
25–29	102	40.80					
30–34	31	12.40					
35–39	17	6.80				Maximum	12
40–44	20	8.00	Female	103	41.20		
45–49	13	5.20					
50–54	6	2.40				Mean	8.15
55–59	1	0.40					
**Total**	**250**	**100**	**Total**	**250**	**100**		

#### Preliminaries

As part of the preliminary analysis, the study explored the intercorrelation that exists between the variables. Table 2 presents the results of the correlation between the variables involved in the study and their means and standard deviation. Newcomer adjustment was negatively correlated with work-related anxiety and turnover intentions (*r* = −0.30, *p* < 0.01; *r* = −0.42, *p* < 0.01, respectively). Separation correlated positively with work-related anxiety and turnover intentions (*r* = 0.81, *p* < 0.01; *r* = 0.64, *p* < 0.01, respectively), however, the correlation between separation and newcomer adjustment was not significant (*r* = −0.11, *p* = 0.072). The relationship between integration and work-related anxiety was negative (*r* = −0.40, *p* < 0.01), and that between integration and turnover intention was negatively correlated (*r* = −0.48, *p* < 0.01). The relationship between integration and adjustment is positively correlated (*r* = 0.94, *p* < 0.01). Even though a high correlation is identified between integration and adjustment, using a tolerance factor of 0.1, the variance inflation factors for both variables were within range.

**TABLE 2:** Descriptive statistics and intercorrelations among variables (*N* = 250).

**Variables**	**1**	**2**	**3**	**4**	**5**
(1) Work-related anxiety	1				
(2) Turnover intention	0.41**	1			
(3) Adjustment	−0.30**	−0.42**	1		
(4) Separation	0.81**	0.64**	–0.11	1	
(5) Integration	−0.40**	−0.48**	0.94**	−0.29**	1
Mean	15.98	8.20	64.40	9.51	14.72
*SD*	6.22	2.86	10.48	2.96	3.72

**Correlation is significant at 0.01.

#### Moderation Analysis

The initial output revealed that all five predictors combined significantly predicted the dependent variable, work-related anxiety, *F*(5,244) = 543.22, *p* < 0.01. Further, the predictors accounted for approximately 92% of the variance in the dependent variable, *R*^2^ = 0.96.

From the PROCESS output presented in Table 3, H1_a_ *b* = 0.09, *t*(244) = 16.74, *p* < 0.01; and H1_b_ *b* = −0.10, *t*(244) = −10.74, *p* < 0.01. Both interactions were statistically different from zero, which means both integration and separation functioned as moderators on the effect of newcomer adjustment on work-related anxiety. It can be observed from Table 4 that the moderation by integration uniquely accounts for 10% of the variance [*F*(1,244) = 280.19, *p* < 0.01], whereas the moderation by separation uniquely accounts for 4% of the variance [*F*(1,244) = 115.26, *p* < 0.01]. The results support both hypotheses H1_a_ and H1_b_, which state that “integration moderates the relationship between adjustment and work-related anxiety, and separation moderates the relationship between adjustment and work-related anxiety,” respectively.

**TABLE 3:** Moderation model for study 1 (work-related anxiety).

	**Coefficient**	***SE***	***t***	***p***	**LLCI**	**ULCI**
Constant	9.18	5.71	81.61	0.109	–2.06	20.42
Adjustment	–0.95	0.1	–8.51	0.000	–1.16	–0.73
Integration	–4.08	0.32	–12.74	0.000	–4.71	–3.45
Int_1	0.09	0.01	16.74	0.000	0.08	0.10
Separation	10.21	0.60	16.95	0.000	9.02	11.39
Int_2	–0.10	0.01	–10.74	0.000	–0.12	–0.08

**TABLE 4:** *R*^2^ increase due to interaction (work-related anxiety).

	***R*^2^ change**	***F***	**df1**	**df2**	***p***
*X* * *W*	0.10	280.19	1.00	244.00	0.000
*X* * *Z*	0.04	115.26	1.00	244.00	0.000

*Predictor: adjustment (X); moderators: integration (W) and separation (Z).*

The study further tested the moderation effect of integration and separation on the relationship between newcomer adjustment and turnover intention. All five predictors jointly significantly predicted the dependent variable, turnover intention, *F*(5,244) = 79.33, *p* < 0.01. The predictors accounted for approximately 62% of the variance in turnover intention, *R*^2^ = 0.62.

**FIGURE 2:**
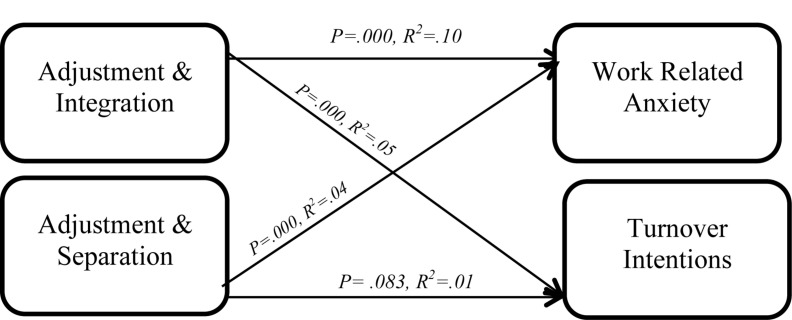
Moderation interaction for study 1.

The result indicated the interaction between newcomer adjustment and integration was significant, H2_a_ *b* = −0.03, *t*(244) = −5.65, *p* < 0.01. On the other hand, the interaction between adjustment and separation on turnover intentions was not significant H2_b_ *b* = 0.02, *t*(244) = 1.74, *p* = 0.083 (Table 5). Moderation by integration exclusively accounted for 5% of the variance [*F*(1,244) = 31.87, *p* < 0.01], whereas the moderation by separation made up for 1% of the variance in the dependent variable [*F*(1,244) = 3.04, *p* = 0.083] (Table 6). The hypothesis stating integration will moderate the relationship between newcomer adjustment and turnover intentions was supported. However, the results did not support the hypothesis stating that separation will moderate the relationship between adjustment and turnover intention. Figure 2 indicates the moderation interaction.

**TABLE 5:** Moderation model with turnover intention as dependent variable.

	**Coefficient**	***SE***	***t***	***p***	**LLCI**	**ULCI**
Constant	2.76	5.64	0.49	0.626	–8.35	13.86
Adjustment	0.13	0.11	1.21	0.227	–0.08	0.35
Integration	1.84	0.32	5.81	0.000	–1.22	2.46
Int_1	–0.03	0.01	–5.65	0.000	–0.04	–0.02
Separation	1.00	0.60	–1.69	0.093	–2.18	0.17
Int_2	0.02	0.01	1.74	0.083	–0.00	–0.03

**TABLE 6:** *R*^2^ increase due to interaction (turnover intentions).

	***R*^2^ change**	***F***	**df1**	**df2**	***p***
*X* * *W*	0.05	31.87	1.00	244.00	0.000
*X* * *Z*	0.01	3.04	1.00	244.00	0.083

*Predictor: adjustment (X); moderators: integration (W) and separation (Z).*

### Study 2

The purpose of study 2 was to go a step further by classifying the sample into two groups considering their immediate past engagement. The researchers attempted to find out if new employees who, prior to their current roles, were in another employment showed any difference compared to newcomers who had come directly from school into their new roles.

Study 2 splits respondents into two different groups, StWEs and WtWEs. The number of respondents for StWEs was 127, of which 57.50% were males (Table 7). Considering their age range, majority (52.80%) of them ranged from 25 to 29 years, and 40.20% ranged between 18 and 24 years. The average period spent in current role was 8 months. For WtWEs, 123 respondents were involved in the study. The majority (60.20%) of these respondents were male, and 28.50% of the total respondents were 25–29 years old. The average number of months respondents who had worked in their current role was approximately 8 months.

**TABLE 7:** Demographic characteristics (study 2).

	**Age**			**Sex**			**Months in current role**	
	**Profile**	**No.**	**%**	**Profile**	**No.**	**%**		
StWE	18–24	51	40.20	Male	73	57.50	Minimum	6
	25–29	67	52.80	Female	54	42.50	Maximum	12
	30–34	9	7.10				Mean	8.25
	Total	127	100	Total	127	100		
WtWE	18–24	9	7.30	Male	74	60.20	Minimum	6
	25–29	35	28.50					
	30–34	22	17.90					
	35–39	17	13.80				Maximum	12
	40–44	20	16.30	Female	49	39.80		
	45–49	13	10.60					
	50–54	6	4.90				Mean	8.05
	55–59	1	0.80					
	**Total**	**123**	**100**	**Total**	**123**	**100**		

#### Preliminaries

Among WtWEs, the relationships between newcomer adjustment and the dependent variables, work-related anxiety and turnover intention, correlated significantly *r* = −0.29, *p* < 0.01; *r* = −0.39, *p* < 0.01, respectively (Table 8). Separation significantly correlated with work-related anxiety and turnover intentions, *r* = 0.79, *p* < 0.01, and *r* = 0.63, *p* < 0.01, respectively, but was not significantly related to newcomer adjustment *r* = −0.10, *p* = 0.282. Further, integration significantly correlated with work-related anxiety, turnover intention, and adjustment, *r* = −0.39, *p* = 0.000; *r* = −0.50; *p* < 0.01, and *r* = 0.95, *p* < 0.01, respectively. As presented in Table 8, the relationships between all the variables among StWEs were significant but for the relationship between adjustment and separation.

**TABLE 8:** Descriptive statistics and intercorrelations (*N* = 123).

**Variables**	**1**	**2**	**3**	**4**	**5**
**WtWE**					
(1) Work-related anxiety	1				
(2) Turnover intention	0.34**	1			
(3) Adjustment	−0.29**	−0.39**	1		
(4) Separation	0.79**	0.63**	–0.10	1	
(5) Integration	−0.39**	−0.50**	0.95**	−0.29**	1
Mean	15.98	8.26	64.42	9.55	14.49
*SD*	6.61	2.90	10.89	3.07	3.84
**StWE**					
(1) Work-related anxiety	1				
(2) Turnover intention	0.49**	1			
(3) Adjustment	−0.31**	−0.46**	1		
(4) Separation	0.83**	0.64**	–0.13	1	
(5) Integration	−0.43**	−0.45**	0.94**	0.29**	1
Mean	15.98	8.14	64.39	9.47	14.95
*SD*	5.85	2.84	10.11	2.87	3.59

**Correlation is significant at 0.01.

#### Moderation Analysis

##### Work-to-work employees

The five interactions significantly predicted the dependent variable, *F*(5,117) = 43.41, *p* < 0.01, accounting for 65% of the variance in the dependent variable, *R*^2^ = 0.65. The results show that the moderation interaction between newcomer adjustment and integration on turnover intentions, with integration serving as the moderator, was significant H3_a_ *b* = −0.03, *t*(117) = −5.65, *p* < 0.01 (Table 9). The interaction between newcomer adjustment and separation with separation as the moderator was also significant H3_b_ *b* = 0.047, *t*(117) = 1.74, *p* < 0.01. Both interactions distinctively made up for 3% of the variance in turnover intention, *R*^2^ = 0.03 (Table 10). These findings support both hypotheses H3_a_ and H3_b_—integration and separation will each moderate the relationship between adjustment and turnover intentions, respectively.

**TABLE 9:** Moderation model with turnover intentions as dependent variable (WtWE).

	**Coefficient**	***SE***	***t***	***p***	**LLCI**	**ULCI**
Constant	16.46	9.06	1.82	0.072	–1.48	34.40
Adjustment	0.05	0.16	0.29	0.775	–0.27	0.37
Integration	1.10	0.57	5.81	0.054	–0.02	2.23
Int_1	–0.03	0.01	–5.65	0.003	–0.04	–0.01
Separation	–3.16	0.93	–1.69	0.001	–4.99	–1.33
Int_2	0.05	0.02	1.74	0.002	0.02	0.08

**TABLE 10:** *R*^2^ increase due to interaction: WtWE (turnover intentions).

	***R*^2^ change**	***F***	**df1**	**df2**	***p***
*X* * *W*	0.03	9.45	1.00	117.00	0.003
*X* * *Z*	0.03	10.56	1.00	117.00	0.002

*Predictor: adjustment (X); moderators: integration (W) and separation (Z).*

**FIGURE 3:**
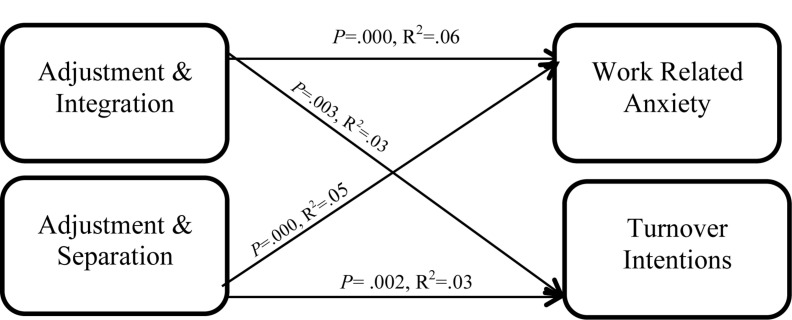
Moderation interaction for work-to-work employees.

Using work-related anxiety as a dependent variable, all assumptions within the model significantly predicted work-related anxiety, *F*(5,117) = 341.34, *p* < 0.01, accounting for 94% of the variance in the dependent variable, *R*^2^ = 0.94. From the results, H4_a_ *b* = 0.09, *t*(117) = 10.39, *p* < 0.01 and H4_b_ *b* = −0.13, *t*(117) = −9.02, *p* < 0.01 (Table 11). Both interactions are statistically different from zero, as such supporting H4_a_ and H4_b_. Observing from Table 12, the interactions involving integration and separation accounted for 6%, *R*^2^ = 0.06, and 5%, *R*^2^ = 0.05, of the variance in work-related anxiety, respectively. Figure 3 shows the results from the interaction among the variables.

**TABLE 11:** Moderation model with work-related anxiety as dependent variable for study 2 (WtWE).

	**Coefficient**	***SE***	***t***	***p***	**LLCI**	**ULCI**
Constant	–3.32	8.83	–0.38	0.708	–20.81	14.18
Adjustment	–0.90	0.16	–5.70	0.000	–1.21	–0.59
Integration	–3.20	0.55	–5.79	0.000	–4.29	–2.10
Int_1	0.09	0.01	10.39	0.000	0.07	0.10
Separation	12.20	0.90	13.52	0.000	10.42	13.98
Int_2	–0.13	0.01	–9.02	0.000	–0.16	–0.10

**TABLE 12:** *R*^2^ increase due to interaction: WtWE (work-related anxiety).

	***R*^2^ change**	***F***	**df1**	**df2**	***p***
*X* * *W*	0.06	10.90	1.00	117.00	0.000
*X* * *Z*	0.05	81.33	1.00	117.00	0.000

*Predictor: adjustment (X); moderators: integration (W) and separation (Z).*

##### School-to-work employees

The predictions with the model significantly predicted the dependent variable, turnover intention, *F*(5,121) = 43.97, *p* < 0.01, resulting in approximately 65% of the variance in the dependent variable, *R*^2^ = 0.65. The interaction between newcomer adjustment and integration was significant, H5_a_ *b* = −0.023, *t*(121) = −3.12, *p* < 0.01, whereas the interaction between newcomer adjustment and separation on turnover intentions was not significant H5_b_ *b* = −0.00, *t*(121) = −0.184, *p* = 0.854 (Table 13). The former contributed 3% of the variance in the dependent variable *R*^2^ = 0.03 (Table 14). On the other hand, the latter does not contribute to the variance in the dependent variable, turnover intention. In this light, the hypothesis stating a moderation effect of integration on the relationship between newcomer adjustment and turnover intention was supported, whereas the hypothesis stating separation will moderate the relationship between newcomer adjustment and turnover intentions was not supported.

**TABLE 13:** Moderation model with turnover intentions as dependent variable (StWE).

	**Coefficient**	***SE***	***t***	***p***	**LLCI**	**ULCI**
Constant	–0.10	7.35	–0.01	0.989	–14.65	14.45
Adjustment	0.03	0.15	0.19	0.848	–0.27	0.33
Integration	1.76	0.39	4.50	0.000	–0.99	2.54
Int_1	–0.02	0.01	–3.12	0.002	–0.04	–0.01
Separation	0.44	0.79	0.56	0.575	–1.11	2.00
Int_2	–0.00	0.01	–0.18	0.854	–0.03	0.02

**TABLE 14:** *R*^2^ increase due to interaction (StWE).

	***R*^2^ change**	***F***	**df1**	**df2**	***p***
X*W	0.03	9.75	1.00	121.00	0.002
X*Z	0.00	0.03	1.00	121.00	0.854

*Predictor: adjustment (X); moderators: integration (W) and separation (Z).*

**FIGURE 4:**
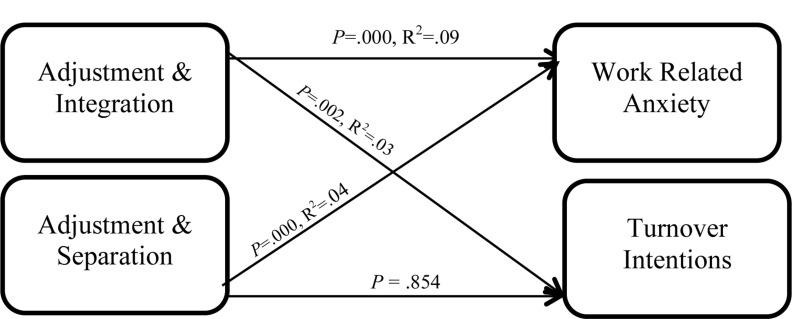
Moderation interaction for school-to-work employees.

All five predictors collectively contributes approximately 91% of the variance in the dependent variable, *R*^2^ = 0.91. Further, the relationship was a significant one, *F*(5,121) = 241.19, *p* < 0.01. All two interactions, the moderation by integration and separation, were significant, H6_a_ *b* = −0.08, *t*(121) = 10.91, *p* < 0.01, and H6_b_ *b* = −0.09, *t*(121) = −7.05, *p* < 0.01, respectively (Table 15). The interactions contributed 9%, *R*^2^ = 0.09 (integration) and 4% (separation) of the variance in work-related anxiety (Table 16). Both hypotheses H6_a_ and H6_b_ were supported by our findings. Figure 4 represents the results from the interaction among the variables.

**TABLE 15:** Moderation model with work-related anxiety as the dependent variable (StWE).

	**Coefficient**	***SE***	***t***	***p***	**LLCI**	**ULCI**
Constant	8.88	7.69	1.16	0.250	–6.34	24.10
Adjustment	–0.79	0.16	–5.01	0.000	–1.10	–0.48
Integration	–3.95	0.41	–9.64	0.000	–4.76	–3.14
Int_1	–0.08	0.08	10.91	0.000	0.07	0.10
Separation	9.17	0.82	11.15	0.000	7.54	10.80
Int_2	–0.09	0.01	–7.05	0.000	–0.11	–0.06

**TABLE 16:** *R*^2^ increase due to interaction (StWE).

	***R*^2^ change**	***F***	**df1**	**df2**	***p***
*X* * *W*	0.09	1119.12	1.00	121.00	0.000
*X* * *Z*	0.04	49.71	1.00	121.00	0.000

*Predictor: adjustment (X); moderators: integration (W) and separation (Z).*

## General Discussion

The purpose of this study was to assess the moderation effect of integration and separation as acculturation strategies on the relationship between newcomer adjustment and turnover intentions and work-related anxiety among newcomers working in Ghana’s private sector, specifically the banking and insurance industries. The results indicate the relationship between newcomer adjustment and work-related anxiety among employees in general is moderated by the acculturation strategy of integration and separation, which is similar to the findings of Finstad et al. (2019). The strength of the relationship between new employees’ adjustment during their second 6 months of work and their level of anxiety resulting from their work is influenced by their ability to be able to combine acquired culture (work-related values, attitudes, principles, etc.) and their new host’s (current organization) culture. Similarly, deciding to detach oneself from the organizational culture and retaining acquired work-related attitudes and behaviors also influence work-related anxiety resulting from adjustment.

Integration as an acculturation strategy was identified to influence the relationship between newcomer adjustment and turnover intentions. This result is supported by Dalvi and Ganji (2014). When newcomers are confronted with new ways of working, social interactions, and the like that are in contrast with their acquired or previous experience, finding a balance between these two contrasts such that none is abandoned completely tends to have an influence on how their level of adjustment affects their intention to leave. On the other hand, the results from this study did not find support for the assertion that separation will moderate the relationship between newcomer adjustment and turnover intentions. This is in contrast with the findings of Labrague et al. (2017) and Seo and Kim (2017).

Previous studies on newcomer adjustment (Lu and Tjosvold, 2013; Gkorezis et al., 2016; Ellis et al., 2017; Oh, 2018) have not taken into consideration the immediate institution the employee is coming; as such, the second part of this study separated participants into two groups, those coming directly from a working role and those directly from school. The framework for the study remained the same as the moderation role of integration and separation was assessed in the relationship between newcomer adjustment and work-related anxiety and turnover intentions. The results from the PROCESS outputs for both samples, WtWEs and StWEs, showed integration moderated the relationship between newcomer adjustment and work-related anxiety. This finding parallels the assertion of Finstad et al. (2019). In support to the findings, Berry and Kim (1988) and Berry (1997) asserted for acculturative stress; there is a clear picture that the pursuit of integration is least stressful, indicating that in the adjustment process an individual’s adoption of integration clearly affects the level of work-related anxiety.

Irrespective of previous experience, the new employees’ ability to employ integration as a strategy to maneuver their way in their new environment has some influence on their level of work-related anxiety. According to Mortensen (2014), work-related anxiety can distort an employee’s performance; as such, it stands to reason that new employees will want to apply the integration strategy in an attempt to reduce their level of anxiety so as to be better performers on the job. The results further supported the claim that separation will moderate the relationship between newcomer adjustment and work-related anxiety in both WtWEs and StWEs, which is affirmed by Sahebazzamani et al. (2009) and Finstad et al. (2019). Notwithstanding whether they are from school to work or from work to work, the effect of successful adjustment or otherwise on newcomers’ turnover intentions is influenced by their ability to employ the acculturation strategy of integration. Finally, as noted earlier, among the general workforce (newcomers), separation did not show an effect on the relationship between newcomer adjustment and their turnover intentions. This is the same for the subgroup of workforce (newcomers) who prior to their current role were students (StWEs), contrary to the assertions of Labrague et al. (2017) and Seo and Kim (2017). However, separation among the sample of workforce who were in active employment before their current role (WtWEs) was identified to influence the relationship between newcomer adjustment and turnover intentions, which is confirmed by Labrague et al. (2017) and Seo and Kim (2017). The insignificance of separation as a moderator could be attributed to the fact that because of the high unemployment rate (Baah-Boateng, 2018), this group of workforce who are just migrating from school to work would not attempt adopting this strategy because of fear of losing their jobs. The other side of the coin could also be that they have not yet concretely acquired any work-related attitudes; as such, this strategy would not be an option for them.

## Conclusion

Organizations will continue to experience both entry and exit of employees as long as it exists, but the rate at which this happens is what they can manage, and how the early stages of the entrants are handled plays a key role. Different socialization theories and adjustment theories have been studied in an attempt to understand the process and its effect on organizational and individual behavioral outcomes. Among immigrants, the acculturation theory has gained prominence in understanding how they adjust to their host culture. In this vein, a similarity could be possible with new employees’ movement into organizations whose operations and culture could be entirely different from their previous firms and as such needs to find a way to settle in. It is in this light that it becomes worthwhile to explore the components of the acculturation strategy within the corporate setting. Specifically, the study sought to introduce the acculturation strategy of integration and separation into the corporate setting by assessing their moderating role on the impact of newcomer adjustment on work-related anxiety and turnover intentions.

A contribution this study offers is the separation of newcomers into StWEs and WtWEs. No previous research has considered new employees based on these classifications. Categorizing new employees into these two groups will allow employers design programs targeted at acquired desirable or undesirable work-related attitudes. The central contribution of this study is in the introduction of acculturation strategies into the socialization process of new employees. These strategies, integration and separation, represent the orientation new employees decide to adopt as they go through the entire socialization process. It has been noted that both strategies have significant influence on adjustment and the level of anxiety experienced during the first 12 months in their new environment. Separation influences the relationship between newcomer adjustment and turnover among WtWEs; however, this was not the case for StWEs and the general sample. This further indicates employers are more likely to lose their WtWEs if they decide to retain their acquired work-related behaviors and reject that of their new environment as a result of the contrast between the two. The kind of acculturation strategy individuals employ has an influence on how their adjustment affects certain behavioral outcomes and as such should not be swept under the carpet. Among the four strategies John Berry identified, the two researched in this study, integration and separation, both have shown to influence the adjustment process. The subject of acculturation is less known in the organizational psychology circles, and this study is set to arouse interest in the application of acculturation theory in understanding employee socialization in the corporate setting. This study touches just a speck of a bigger spectrum.

## Recommendations

Rudmin (2003) asserted that the level of difficulty versus easiness involved in integrating an immigrant’s host culture and home culture, in part, is determined by the degree of similarities between both cultures. In this light, this study proposes that further studies could explore the degree of similarities between the new employee’s previous organizational culture and current organizational culture. A variable that might be important to consider is whether StWEs have ever worked before, either as an intern or before going to school. This variable could play a role in their adjustment process and is worth studying. Finally, it will be interesting to explore whether the kind of socialization tactics employed by the organization will influence the acculturation strategy the individual adopts, considering some of the tactics, for example, investiture and divestiture, are geared toward affirming and undoing, respectively, certain attributes of these new employees.

## Theoretical Implications

The subject of acculturation is less known in organizational psychology circles, and this study is set to arouse interest in the application of acculturation theory in understanding employee socialization. The period of socialization for new employees is in no doubt synonymous to the period of adaptation immigrants go through. As such, it is not out of place to adopt the one theory that is predominantly used in understanding immigrants’ adaptation to new employees’ adaptation. This study as such becomes just the tip of the iceberg. The results of this study will serve as a basis for theoretical debate on acculturation theory in corporate institutions.

## Practical Implications

The findings from this study will help newcomers in organizations to be mindful of how they handle cultural differences and the position they choose to adopt as it has the ability to affect their level of work-related anxiety. It is recommended that, in designing of the socialization program for newcomers, a consideration be given to the fact that these new employees are coming with some acquired behaviors, as such, how it will be handled and whether the organization will want them to maintain them and find a blend between the “host culture” and the “home culture” or otherwise should be incorporated in the program. Finally, the orientation program should not be entirely generalized as StWEs and WtWEs reacted differently.

## Limitations of the Study

Notwithstanding all the necessary precautions adopted, this study, just like any other study, had some limitations. The main limitation of the study is the use of cross-sectional design as it only points out the relationship between the variables. However, the use of this design has raised key issues that would be the foundation for a longitudinal study. Further studies are advised to control for previous working experience in both groups as this might have an effect on the relationships. This was noted as another limitation of the study. Again, the data gathered for this study adopted a single means, questionnaire. A combination of different methods will shed more light on the study area.

## Data Availability Statement

The datasets generated for this study are available on request to the corresponding author.

## Ethics Statement

The studies involving human participants were reviewed and approved by Zhejiang University, Department of Psychology and Behavioral Sciences. Written informed consent for participation was not required for this study in accordance with the national legislation and the institutional requirements.

## Author Contributions

CH and JM: conceptualization. CH: data gathering, analysis, and writing – original draft. JM: supervision. LA and PH: reviewing of draft and analysis. All authors contributed to the article and approved the submitted version.

## Conflict of Interest

The authors declare that the research was conducted in the absence of any commercial or financial relationships that could be construed as a potential conflict of interest.
